# The Systemic Immune–Inflammation Index Predicts Long-Term Outcomes in Patients With Unstable Angina and Diabetes After Revascularization

**DOI:** 10.31083/RCM36261

**Published:** 2025-08-15

**Authors:** Xiaowen Bo, Tian Zhou, Hao Zhang, Siyuan Chen, Ning Yin, Donghui Zhao, Shujuan Cheng, Jinghua Liu, Qian Fan

**Affiliations:** ^1^Department of Cardiology, Beijing AnZhen Hospital, Capital Medical University, Beijing Institute of Heart, Lung, and Blood Vessel Diseases, 100029 Beijing, China

**Keywords:** unstable angina, diabetes mellitus, systemic immune-inflammation index

## Abstract

**Background:**

The incidence of unstable angina (UA), a type of cardiovascular disease (CVD), has increased in recent years. Meanwhile, timely percutaneous coronary intervention (PCI) or percutaneous transluminal coronary angioplasty (PTCA) procedures are crucial for patients with UA who also have diabetes mellitus (DM). Additionally, exploring other factors that may influence the prognosis of these patients could provide long-term benefits. The systemic immune-inflammation index (SII), a novel marker for assessing inflammation levels, has been shown to correlate with the long-term prognosis of various diseases. Thus, this study aimed to investigate the predictive value of the SII for the long-term prognosis of patients with UA and DM after revascularization.

**Methods:**

A total of 937 UA patients who underwent revascularization, of which 359 also had DM, were included in this study. Patients were divided into two groups: the low SII group (<622.675 × 10^9^/L; n = 219, 61.0%) and the high SII group (≥622.675 × 10^9^/L; n = 140, 39.0%). The primary outcome was the frequency of major adverse cardiovascular and cerebrovascular events (MACCEs). The secondary outcome was the incidence of all-cause death.

**Results:**

Of the 359 patients who visited our institution between January 2018 and January 2020, 23 patients (10.5%) in the low SII group experienced MACCEs, whereas 34 cases (24.3%) in the high SII group experienced MACCEs, showing a statistically significant difference (*p* < 0.001). After conducting univariate and multivariate regression analyses on the endpoint events, we identified several risk factors for MACCEs. These risk factors included high SII levels, a history of myocardial infarction (MI), prior PCI or coronary artery bypass grafting (CABG), elevated brain natriuretic peptide (BNP), and the lack of angiotensin-converting enzyme inhibitors (ACEI) or statin use. Upon adjusting for covariates including age, sex, body mass index (BMI), BNP, smoking, hypertension, PCI or CABG history, MI history, statin use, ACEI use, and the presence of three-vessel coronary disease, only high SII levels remained a risk factor for MACCEs (HR: 0.155, 95% CI: 0.063–0.382; *p* = 0.001). However, high SII levels were not identified as a risk factor for other individual endpoint events, including non-fatal stroke, cardiovascular death, non-fatal MI, or cardiac rehospitalization.

**Conclusion:**

Elevated SII levels following percutaneous intervention are associated with poor outcomes in patients with UA and DM. Therefore, regular monitoring and controlling inflammation levels may help improve long-term outcomes.

## 1. Introduction

Cardiovascular disease (CVD) is the most common cause of mortality and morbidity worldwide [1, 2]. Acute coronary syndrome (ACS) is the most common clinical manifestation of CVD, with an estimated 5.8 million new cases of ischemic heart disease worldwide in 2019 [1]. Unstable angina (UA) is another type of ACS that does not involve segment elevation myocardial infarction (STEMI) and non-segment elevation myocardial infarction (NSTEMI). It primarily presents as post-active angina pectoris and is considered a gray area between stable angina pectoris and myocardial infarction (MI) [3]. Diabetes is a metabolic disease characterized by abnormal blood sugar levels [4]. In recent years, the prevalence of diabetes has been rising steadily, making it a serious public health concern [5]. As one of the risk factors for coronary atherosclerosis, diabetes may share a common underlying pathological mechanism related to abnormal systemic inflammation levels [6].

The systemic immune-inflammation index (SII) is calculated by multiplying the platelet count by the neutrophil count and then dividing by the lymphocyte count. It is a new and reliable indicator for comprehensively assessing the inflammation levels in subjects [7]. It has long been recognized that the development and progression of coronary atherosclerotic heart disease are closely linked to inflammation. In recent years, various inflammatory markers have been associated with diabetes. Our research focuses on the long-term prognostic ability of these inflammatory markers in UA patients with diabetes, after receiving percutaneous coronary intervention (PCI) treatment.

## 2. Methods

We evaluated patients with unstable angina who were treated with coronary stenting or balloon angioplasty between January 2018 and January 2020, retrospectively at the Beijing Anzhen Hospital. There were 1190 consecutive patients diagnosed with UA, based on their clinical characteristics, laboratory results, and electrocardiograph [8, 9]. We excluded patients who self-reported inflammatory diseases such as pneumonia, cystitis, and pharyngitis, as well as those on medications, including antibiotics, hormones, or treatments for autoimmune diseases. Based on their complete laboratory test results, we included a total of 937 patients, among which 359 diabetic patients were included in this study. After enrollment, trained nurses and cardiologists collected data on PCI procedures and treatment strategies. Baseline characteristics were recorded, which included comorbid conditions, laboratory tests, echocardiography results, personal history, lesion characteristics, medication, and other laboratory data. The comorbid conditions include hypertension, diabetes, chronic heart failure, dyslipidemia, previous myocardial infarction, and previous PCI or coronary artery bypass grafting (CABG). Laboratory examination assessed renal function, lipid profile, and hemograms at enrolment as a medical record. The calculation for SII is as follows: SII equals to total peripheral platelets count (P) × neutrophil-to-lymphocyte ratio (N/L) (SII = P × N/L ratio).

The primary outcome was major adverse cardiac and cerebrovascular events (MACCEs), including a composite of cardiovascular death, non-fatal MI, non-fatal stroke and cardiac rehospitalization. MI was confirmed in patients presenting with ischemic symptoms with elevated serum cardiac enzyme levels and/or characteristic electrocardiogram (ECG) changes. Ischemic stroke was defined as obstruction within a blood vessel supplying blood to the brain with imaging evidence by either magnetic resonance imaging (MRI) or computed tomography (CT) scans and new neurologic deficit lasting for at least 24 hours. Cardiac rehospitalization is defined as any hospital admission that occurs after the initial hospitalization due to cardiac-related issues, including a range of heart-related conditions and complications.

Categorical variables were summarized as numbers (percentages) and compared using the chi-square test or Fisher’s exact test. Continuous variables were compared between groups using one-way analysis of variance (ANOVA) and expressed as the mean and standard deviation in the event of a normal or median distribution and as the interquartile range in the event of an asymmetric distribution. In the case of non-normal distribution, Mann-Whitney U test was used for statistical analysis. The primary and secondary clinical outcomes were presented as overall percentages and expressed as proportions with a 95% confidence interval (CI).

The prognostic difference and event-free survival rate between patients with different SII groups were analyzed using the Kaplan-Meier method, and the significance was evaluated using log-rank tests. Hazard ratios (HRs) for the regression of Cox proportional hazards adjusted with comorbidities and medications were used, along with the corresponding standard error, 95% CI and *p* value. Independent baseline variables with a *p* value < 0.05 in the univariate analysis were included in the multivariate analysis. All statistical analyses were undertaken with SPSS 20.0 software (IBM, Armonk, NY, USA). Furthermore, the study complies with the Declaration of Helsinki, and approval was obtained from the Ethics Committees and Independent Review Boards in Beijing Anzhen Hospital (No.2024145X). All patients signed a written informed consent form to participate in the study prior to any procedures. Reporting of the study conforms to STrengthening the Reporting of OBservational studies in Epidemiology (STROBE) statement along with references to STROBE statement and the broader Enhancing the QUAlity and Transparency Of health Research (EQUATOR) guidelines.

## 3. Results

### 3.1 Baseline and Procedural Characteristics

**Fig. 1.**
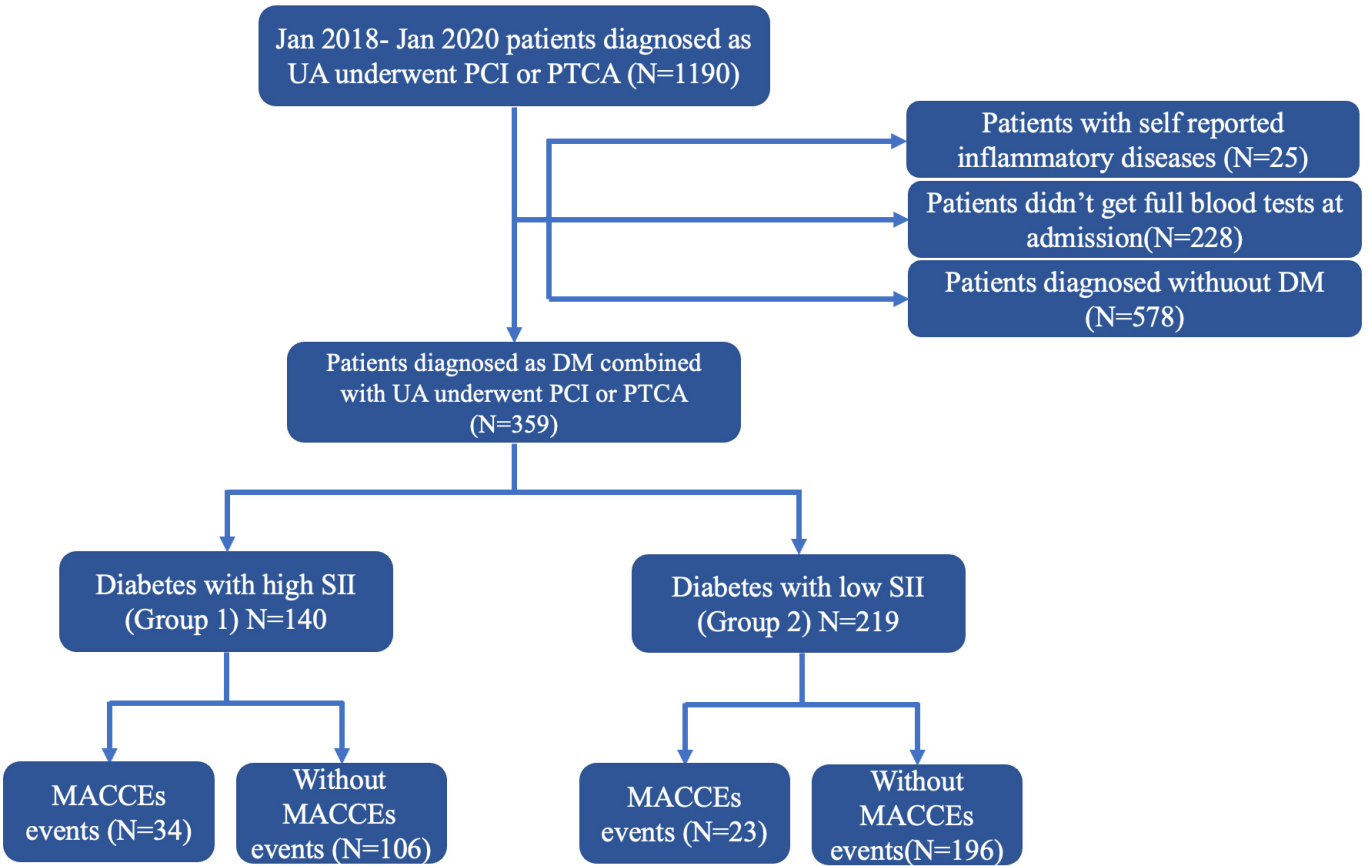
**Flowchart**. UA, unstable angina; PCI, percutaneous coronary intervention; PTCA, percutaneous transluminal coronary angioplasty; DM, diabetes mellitus; SII, systemic immune-inflammation index; MACCEs, major adverse cardiac and cerebrovascular events.

We retrospectively included 937 patients diagnosed with UA based on the inclusion criteria (Fig. 1). Among the 937 patients, 359 (38.3%) were diagnosed with diabetes. The patients were divided into two groups based on their SII levels. Table 1 shows the baseline characteristics of the total population.

**Table 1.** **Baseline clinical characteristics of total population**.

		Total population	Diabetes	Without diabetes	χ ^2^	*p* value
		N = 937	N = 359	N = 578		
Male, n (%)		688 (73.9)	232 (64.6)	456 (78.9)	23.11	<0.001
Age (years)		58.7 ± 12.1	56.9 ± 11.0	60.2 ± 12.9	-	<0.001
Previous or current smoking, n (%)		761 (35.7)	237 (66.0)	524 (90.7)	88.14	<0.001
Hypertension, n (%)		648 (69.2)	264 (73.5)	384 (66.4)	5.24	0.022
Dyslipidemia, n (%)		770 (82.2)	305 (85.0)	465 (80.4)	3.07	0.080
Previous MI, n (%)		23 (2.5)	8 (2.2)	15 (2.6)	0.12	0.724
Previous PCI or CABG, n (%)		184 (19.6)	83 (23.1)	101 (17.5)	4.47	0.034
Laboratory data						
	LDL-C (mmol/L)	2.1 ± 0.8	2.0 ± 0.8	2.2 ± 0.8	-	<0.01
	hsCRP (mg/L)	1.0 (0.6–2.4)	1.0 (0.6–2.5)	1.0 (0.5–2.4)	-	0.591
	hs‐TnI	4.9 (2.7–10.6)	4.8 (2.7–11.6)	4.9 (2.6–10.0)	-	0.623
	SII	573.6 (402.9, 781.1)	559.8 (397.3, 798.2)	576.2 (407.9, 773.5)	-	0.356
Medication						
	Aspirin, n (%)	937 (100)	359 (100)	578 (100)	-	ns
	Clopidogrel or ticagrelor, n (%)	937 (100)	359 (100)	578 (100)	-	ns
	Statin, n (%)	931 (99.4)	354 (98.6)	577 (99.8)	5.18	0.023
	ACEI/ARB, n (%)	736 (78.5)	292 (81.3)	444 (76.8)	2.67	0.101
	β‐blockers, n (%)	788 (84.1)	306 (85.2)	482 (83.5)	0.56	0.453
Lesion characteristic						
	One‐vessel disease, n (%)	308 (32.9)	108 (30.1)	200 (34.6)	2.05	0.152
	Two‐vessel disease, n (%)	405 (43.2)	162 (45.1)	243 (42.2)	0.86	0.354
	Three‐vessel disease, n (%)	224 (23.9)	89 (24.8)	135 (23.4)	0.251	0.617
	Chronic total occlusion, n (%)	126 (13.4)	45 (12.4)	81 (14.0)	0.42	0.519
MACCEs, n (%)		152 (16.2)	57 (15.9)	95 (16.4)	0.05	0.822
	Non-fatal stroke, n (%)	20 (2.1)	8 (2.2)	12 (2.1)	0.03	0.875
	Cardiovascular death, n (%)	15 (1.6)	6 (1.7)	9 (1.6)	0.02	0.892
	Non-fatal MI, n (%)	49 (5.2)	17 (4.7)	32 (5.5)	0.29	0.592
	Cardiac rehospitalization, n (%)	68 (7.3)	26 (7.2)	42 (7.3)	0.00	0.989

LDL-C, low-density lipoprotein cholesterol; hsCRP, hypersensitive C-reactive protein; hs-TnI, high-sensitivity troponin I; SII, systemic immune-inflammation index; ACEI, angiotensin converting enzyme inhibitors; ARB, angiotonin receptor blocker; MACCEs, major adverse cardiac and cerebrovascular events; MI, myocardial infarction; CABG, coronary artery bypass grafting; PCI, percutaneous coronary intervention; ns, no significance.

We observed that there was no significant difference in the proportion of males and age between the two groups of people. The proportion of smokers in the high SII level population was higher than that in the low SII level population, but the body mass index (BMI) value was significantly lower than that in the low SII level population (*p* < 0.05). Regarding comorbidities, for hypertension and hyperlipidemia, there was no significant difference between the two groups. However, the previous MI and previous PCI or CABG cases in the low SII level group were higher than those in the high SII level group (*p* < 0.05). In terms of laboratory tests, the low-density lipoprotein cholesterol (LDL-C) level in the low SII level group was higher than that in the high SII level group, and the hypersensitive C-reactive protein (hsCRP) and high-sensitivity troponin I (hs-TnI) levels were both lower than those in the high SII level group (*p* < 0.05). There were no significant differences between the two groups in other laboratory tests. There were no significant differences in medication use and lesion characteristics between the two groups (*p* > 0.05) (Table 2).

**Table 2.** **Clinical characteristics of UA combined with DM patients underwent PCI or PTCA**.

		Diabetes with high SII (Group 1)	Diabetes with low SII (Group 2)	χ ^2^	*p* value
		N = 140	N = 219		
SII		889.8 ± 201.6	475.1 ± 104.8	-	<0.001
Male, n (%)		85 (60.7)	147 (67.1)	1.534	0.215
Age (years)		63.9 ± 10.5	63.8 ± 8.7	-	0.922
Previous or current smoking, n (%)		105 (75.0)	132 (60.3)	8.255	0.004
BMI		25.2 ± 4.3	26.1 ± 3.5	-	0.030
Hypertension, n (%)		108 (77.1)	156 (71.2)	1.533	0.216
Dyslipidemia, n (%)		117 (83.6)	188 (85.8)	0.345	0.557
Previous MI, n (%)		3 (2.1)	5 (2.3)	0.008	0.930
Previous PCI or CABG, n (%)		32 (22.9)	51 (23.3)	0.009	0.925
Laboratory data					
	LDL-C (mmol/L)	1.8 ± 0.8	2.2 ± 0.9	-	<0.001
	hsCRP (mg/L)	1.6 (1.1, 4.0)	0.9 (0.6, 2.3)	-	0.004
	hs‐TnI	22.1 (4.3, 39.8)	4.8 (2.6, 10.2)	-	0.011
	Triglyceride	1.7 (1.4, 2.2)	1.6 (1.0, 1.8)	-	0.760
	Total cholesterol	3.7 (2.7, 3.9)	3.4 (3.1, 4.7)	-	0.136
	Glucose	7.1 ± 2.0	7.3 ± 3.1	-	0.498
	CREA	70.1 (58.4, 104.8)	73.3 (68.9, 87.0)	-	0.860
	eGFR	78.8 ± 18.9	79.4 ± 20.9	-	0.783
	BNP	74 (38.5, 283.5)	70.0 (19.0, 134.5)	-	0.975
	Glycated albumin	18.5 ± 3.4	18.8 ± 4.7	-	0.514
	Glycated hemoglobin	7.6 ± 0.7	7.4 ± 1.4	-	0.117
Medication					
	Aspirin, n (%)	140 (100)	219 (100)	-	ns
	Clopidogrel or ticagrelor, n (%)	140 (100)	219 (100)	-	ns
	Statin, n (%)	138 (98.6)	216 (98.6)	0.002	0.963
	ACEI/ARB, n (%)	110 (78.6)	182 (83.1)	1.156	0.282
	β‐blockers, n (%)	122 (87.1)	184 (84.0)	0.663	0.416
	CCB, n (%)	43 (30.7)	75 (34.2)	0.483	0.487
Lesion characteristic					
	One‐vessel disease, n (%)	36 (25.7)	72 (32.9)	2.083	0.149
	Two‐vessel disease, n (%)	71 (50.7)	91 (41.6)	2.895	0.089
	Three‐vessel disease, n (%)	33 (23.6)	56 (25.6)	0.183	0.669
	Chronic total occlusion, n (%)	20 (14.3)	25 (11.4)	0.642	0.423

BMI, body mass index; MI, myocardial infarction; PCI, percutaneous coronary intervention; CABG, coronary artery bypass grafting; CREA, creatinine; eGFR, estimated glomerular filtration rate; BNP, brain natriuretic peptide; CCB, calcium channel blockers; LDL-C, low-density lipoprotein cholesterol; hsCRP, hypersensitive C-reactive protein; SII, systemic immune-inflammation index; ACEI, angiotensin converting enzyme inhibitors; ARB, angiotonin receptor blocker; MACCEs, major adverse cardiac and cerebrovascular events; MI, myocardial infarction; PTCA, percutaneous transluminal coronary angioplasty; UA, unstable angina; DM, diabetes mellitus; ns, no significance; hs-TnI, high-sensitivity troponin I.

### 3.2 Clinical Endpoint Events After SII Grouping

**Fig. 2.**
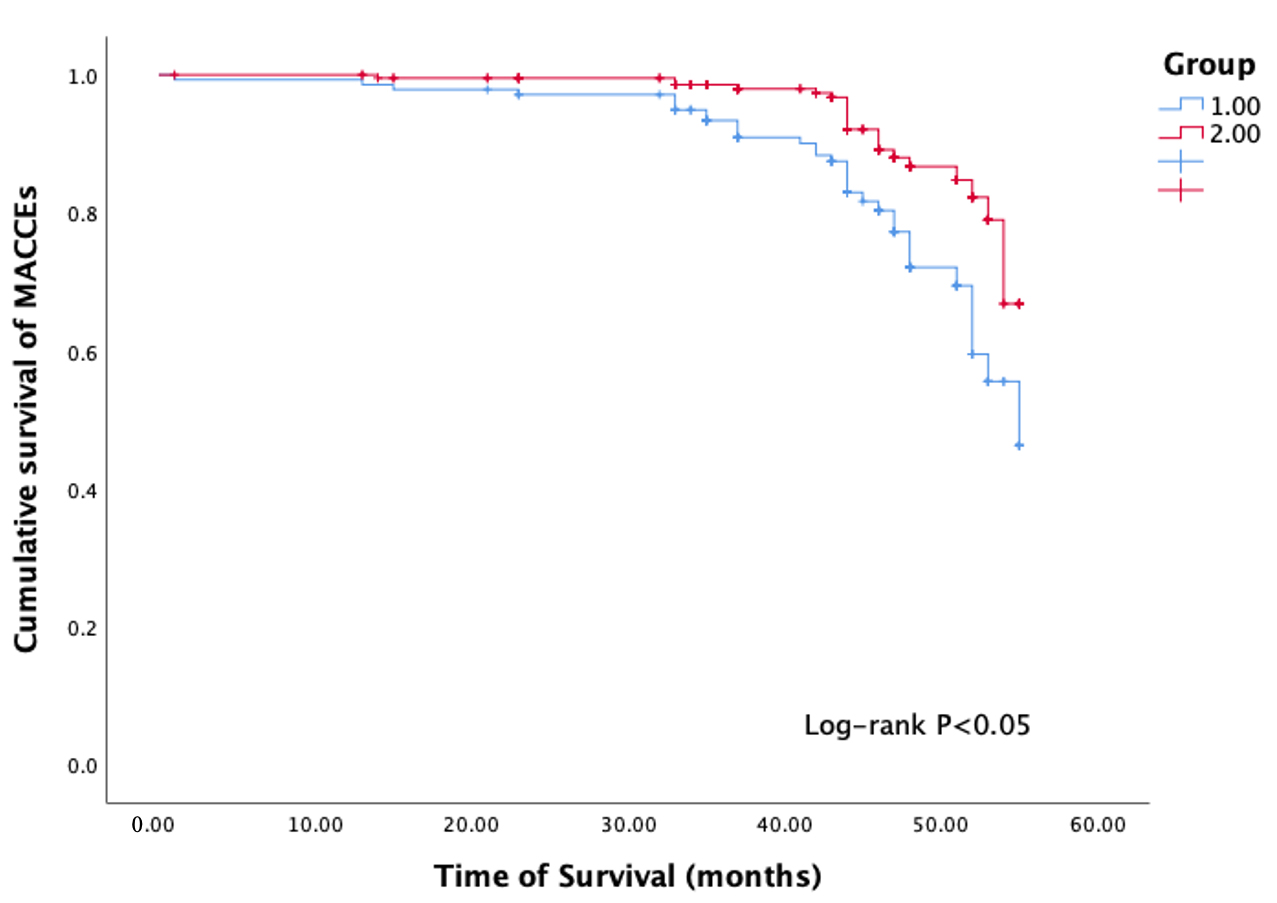
**Kaplan-Meier (KM) survival curve**. Group 1 diabetes with high SII; Group 2 diabetes with low SII; MACCEs, major adverse cardiac and cerebrovascular events; SII, systemic immune-inflammation index.

The average follow-up period was approximately 50 months. Regarding outcomes, there were a total of 57 MACCEs events, 8 cases of non-fatal stroke, 6 cardiovascular deaths, 17 cases of non-fatal myocardial infarction, and 26 hospitalizations due to heart failure (Table 3). Among the 140 subjects with higher baseline SII, 34 cases (24.3%) experienced MACCEs events, 11 cases (7.9%) had non-fatal myocardial infarction, 3 cases (2.1%) had non-fatal stroke, and 16 cases (11.4%) were hospitalized for congestive heart failure. In contrast, the group with low SII had significantly lower incidences of MACCEs events (10.5%), cardiac death (0.9%), non-fatal myocardial infarction (2.7%), and heart failure hospitalization (4.6%) during the follow-up period (Table 3). The Kaplan-Meier curve showed that there was a difference in the occurrence of MACCEs events between the two groups during follow-up (*p* < 0.05) (Fig. 2). This suggests that higher SII is associated with a worse prognosis in UA patients with diabetes mellitus (DM). After performing univariate regression analysis on the occurrence of MACCEs events, we found that higher SII, smoking, or a history of myocardial infarction, prior PCI or CABG, elevated brain natriuretic peptide (BNP), non-use of statins, and non-use of angiotensin converting enzyme inhibitors (ACEI) or angiotonin receptor blocker (ARB), as well as triple-vessel disease, were associated with the occurrence of MACCEs events. In multivariate regression analysis, we found that higher SII, a history of myocardial infarction, previous PCI or CABG, and irregular use of statins and ACEI were related to the occurrence of MACCEs events (Table 4). When analyzing different endpoints, including MACCEs events, non-fatal MI, non-fatal stroke, cardiac rehospitalization, and cardiovascular death, we found that in model 3, only the occurrence of MACCEs events was significantly higher in the high SII group compared to the low SII group, while the other endpoint events did not show a significant increase (Table 5).

**Table 3.** **Clinical endpoint events after SII grouping**.

	Diabetes with high SII (Group 1)	Diabetes with low SII (Group 2)	χ ^2^	*p* value
	N = 140	N = 219		
MACCEs, n (%)	34 (24.3)	23 (10.5)	12.148	<0.001
Non-fatal myocardial infarction, n (%)	11 (7.9)	6 (2.7)	4.958	0.026
Non-fatal stroke, n (%)	3 (2.1)	5 (2.3)	0.000	1.000
Cardiovascular death, n (%)	4 (2.9)	2 (0.9)	0.959	0.327
Cardiac rehospitalization, n (%)	16 (11.4)	10 (4.6)	5.987	0.014

MACCEs, major adverse cardiac and cerebrovascular events; SII, systemic immune-inflammation index.

**Table 4.** **Cox univariate and multivariate analysis of MACCEs events**.

	Univariate OR (95% CI)	*p*-value	Multivariate OR (95% CI)	*p*-value
Group 2 vs 1	0.437 (0.258–0.742)	0.002	0.207 (0.090–0.475)	<0.001
BMI	0.935 (0.864–1.013)	0.099	1.053 (0.939–1.181)	0.373
Gender (female vs male)	1.154 (0.579–1.963)	0.596	NA	NA
Age	1.018 (0.989–1.048)	0.215	NA	NA
Smoking	6.137 (2.850–13.215)	<0.001	1.660 (0.688–4.006)	0.259
Hypertension	1.026 (0.561–1.878)	0.933	NA	NA
Dyslipidemia	1.303 (0.590–2.878)	0.512	NA	NA
Previous MI	6.867 (3.228–14.608)	<0.001	4.154 (1.347–12.808)	0.013
Previous PCI or CABG	9.753 (5.408–17.591)	<0.001	6.918 (2.903–16.485)	<0.001
LDL-C	0.947 (0.669–1.341)	0.759	NA	NA
HDL-C	0.476 (0.153–1.480)	0.200	NA	NA
Triglyceride	1.112 (0.913–1.354)	0.291	NA	NA
Total cholesterol	0.986 (0.759–1.311)	0.986	NA	NA
hsCRP	1.057 (0.965–1.157)	0.234	NA	NA
hs‐TnI	1.001 (1.000–1.001)	0.131	NA	NA
Glucose	0.997 (0.900–1.105)	0.957	NA	NA
CREA	0.987 (0.971–1.004)	0.139	NA	NA
eGFR	1.003 (0.987–1.019)	0.690	NA	NA
BNP	1.001 (1.000–1.003)	0.043	1.002 (1.001–1.003)	0.005
glycated albumin	1.021 (0.959–1.087)	0.513	NA	NA
glycated hemoglobin	0.850 (0.575–1.257)	0.416	NA	NA
Statin	0.092 (0.033–0.258)	0.001	0.190 (0.038–0.941)	0.042
ACEI/ARB	0.160 (0.094–0.270)	0.001	0.430 (0.187–0.990)	0.047
Three‐vessel disease	7.290 (4.088–12.999)	0.001	2.320 (0.930–5.785)	0.071

BMI, body mass index; PCI, percutaneous coronary intervention; CABG, coronary artery bypass grafting; MI, myocardial infarction; CREA, creatinine; eGFR, estimated glomerular filtration rate; BNP, brain natriuretic peptide; LDL-C, low-density lipoprotein cholesterol; HDL-C, high-density lipoprotein cholesterol; hsCRP, hypersensitive C-reactive protein; SII, systemic immune-inflammation index; ACEI, angiotensin converting enzyme inhibitors; ARB, angiotonin receptor blocker; MACCEs, major adverse cardiac and cerebrovascular events; MI, myocardial infarction; NA, not applicable; hs-TnI, high-sensitivity troponin I.

**Table 5.** **Association between SII and adverse events in patients with UA combined with DM after revascularization**.

		Events (n%)	Model 1		Model 2		Model 3	
			HR (95% CI)	*p* value	HR (95% CI)	*p* value	HR (95% CI)	*p* value
MACCEs								
	Group 1	34 (24.3)	Reference	-	Reference	-	Reference	-
	Group 2	23 (10.5)	0.423 (0.249–0.720)	0.002	0.197 (0.088–0.444)	0.001	0.155 (0.063–0.382)	0.001
Non-fatal stroke								
	Group 1	3 (2.1)	Reference	-	Reference	-	Reference	-
	Group 2	5 (2.3)	0.790 (0.211–2.952)	0.726	0.453 (0.065–3.174)	0.425	0.309 (0.034–2.781)	0.295
Cardiovascular death								
	Group 1	4 (2.9)	Reference	-	Reference	-	Reference	-
	Group 2	2 (0.9)	0.314 (0.057–1.713)	0.181	0.227 (0.018–2.880)	0.253	0.023 (0.001–1.273)	0.065
Non-fatal MI								
	Group 1	11 (7.9)	Reference	-	Reference	-	Reference	-
	Group 2	6 (2.7)	0.346 (0.127–0.939)	0.037	0.610 (0.112–3.315)	0.502	0.281 (0.042–1.863)	0.188
Cardiac rehospitalization								
	Group 1	16 (11.4)	Reference	-	Reference	-	Reference	-
	Group 2	10 (4.6)	0.394 (0.178–0.869)	0.021	0.331 (0.102–1.076)	0.066	0.290 (0.080–1.048)	0.059

Notes: Model 1: covariates were adjusted for age and sex. Model 2: covariates were adjusted for age, sex, BMI, BNP, smoking, hypertension. Model 3: covariates were adjusted for age, sex, BMI, BNP, smoking, hypertension, PCI or CABG history, MI history, statin, ACEI, and three-vessel coronary disease. HR, hazard ratio; MACCEs, major adverse cardiac and cerebrovascular events; MI, myocardial infarction; SII, systemic immune-inflammation index; UA, unstable angina; DM, diabetes mellitus; BMI, body mass index; BNP, brain natriuretic peptide; PCI, percutaneous coronary intervention; CABG, coronary artery bypass grafting; ACEI, angiotensin converting enzyme inhibitors.

## 4. Discussion

In this retrospective study, we found that high SII levels may be associated with future MACCEs events, cardiogenic rehospitalization, and non-fatal myocardial infarction in patients with diabetes and unstable angina. After adjusting for risk factors, high SII levels remained consistently associated with MACCEs events.

SII, as a novel inflammatory marker, was first identified by Hu *et al*. [10] in hepatocellular carcinoma, and the index had significant associations with prognostic clinical outcomes, including vascular invasion, tumor size, and early recurrence. With the gradual exploration of this index, it has been increasingly recognized that, in addition to tumors, SII can also serve as a predictor of poor prognosis in diseases such as diabetes and coronary heart diseases. Nie *et al*. [11] conducted an analysis of a large cross-sectional population database in the United States and found that with each additional unit of SII, the likelihood of having diabetes increased by 4% (OR = 1.04; 95% CI: 1.02–1.06; *p* = 0.0006). Cao *et al*. [12] found that according to the National Health and Nutritional Examination Surveys (NHANES) 2011–2018 with a total population of 8524 adults with hypertension, a higher SII (whether as a continuous or categorical variable) was significantly associated with an increased risk of CVD mortality. Similarly, in populations with CVD, there have been comparable study. Previously, Liu *et al*. [13] attempted to predict the severity of coronary stenosis by exploring the levels of inflammatory markers in CVD patients and found that SII was the best indicator for predicting coronary stenosis. Although the population included in this study also consisted of CVD patients, their study did not specify the different types of CVD. In fact, different types of CVD have significant differences in their pathophysiological processes, with unstable angina being one form of CVD.

UA is often defined as myocardial ischemia at rest or on minimal exertion in the absence of acute cardiomyocyte injury/necrosis [14]. The main pathological manifestations were incomplete occlusion of coronary vessels after intravascular plaque rupture [8, 15]. Plaque rupture is associated with the tearing of the endothelial wall, which triggers platelet aggregation and release of particle contents. This process is accompanied by additional platelet aggregation, vasoconstriction, and thrombosis, all of which are significant contributors to UA [16]. Inflammation also plays a crucial role in hemostatic and coagulation pathways. Inflammatory acute-phase reactants, cytokines, chronic infections, and surges of catecholamine can stimulate an increase in tissue factor production, procoagulant activity, and platelet hyperaggregability [17]. These factors promote the formation of incomplete thrombosis and are characteristic of unstable angina [18, 19, 20]. With the increasing incidence of coronary heart disease in recent years, the number of patients with unstable angina pectoris is also increasing [21]. In patients with ACS, including UA, inflammation is the primary driver of myocardial ischemia-reperfusion injury [22, 23]. Additionally, the prevalence of diabetes is also increasing year by year [24]. As a significant risk factor for coronary heart disease [25], high blood sugar levels can damage the vascular endothelium of the coronary arteries [26, 27], and lead to changes in inflammatory markers [28, 29].

As highlighted earlier, there is a growing emphasis on the benefits of reducing residual inflammation risk through various treatments [30, 31, 32, 33]. A substantial body of research has confirmed that controlling inflammation levels significantly improves the prognosis for patients with coronary artery disease. Our study included patients with UA who also had diabetes, representing nearly 40% of the UA population. This is consistent with the current proportion of coronary artery disease patients with diabetes. High levels of inflammation are a common risk factor for both conditions. Therefore, more aggressive control of inflammation levels could have a significant impact on the long-term prognosis of these patients. We found that after adjusting for risk factors using multiple models, the SII remains an adverse prognostic factor for these patients following PCI (HR: 0.155, 95% CI: 0.063–0.382, *p* = 0.001). For patients with high SII levels, who are associated with adverse long-term outcomes, it is crucial to focus on the control and monitoring of inflammation after vascular revascularization. We believe that individualized treatment plans should be prioritized, incorporating dynamic changes in SII to promptly adjust anti-inflammatory therapy and metabolic management strategies. This approach aims to improve long-term prognosis, including the enhancement of multidisciplinary collaboration to optimize the comprehensive management of diabetes and cardiovascular diseases.

However, for patients who have already experienced adverse events such as myocardial infarction, including STEMI or NSTEMI, the ability to improve prognosis by controlling inflammation levels is limited, as necrotic myocardial cells do not regenerate after MI. Despite this, many studies continue to focus on this group of patients. For instance, Liu *et al*. [34] included 216 STEMI patients and conducted blood tests upon admission, 12 hours after PCI, and at discharge. They found that the systemic inflammation response index (SIRI) value at 12 hours post-PCI (HR: 1.079; 95% CI: 1.050–1.108; *p* < 0.001) was independently associated with an increased risk of major adverse cardiovascular events (MACEs). Similarly, Zhu *et al*. [35] followed 355 STEMI patients for one year and found that the SII of patients who experienced MACE events within the year was significantly different from that of the non-MACE group (*p* = 0.003). In a multivariable Cox regression analysis, SII was found to be an independent predictor of long-term MACE (*p* < 0.001, HR: 2.952, 95% CI: 1.565–5.566).

Similar studies have been conducted in patients with NSTEMI. Yaşan *et al*. [36] included 28 patients who underwent coronary angiography due to NSTEMI. Patients were divided into three strata based on SII levels. The relationship between SII levels and 1-year, 3-year, and 5-year mortality rates (NSTEMI) was studied. At various follow-up time points, higher SII levels were found to be associated with increased mortality. Compared to the lower and middle tertiles of SII, the 1-year mortality rate was significantly higher in patients in the upper SII tertile [11 (15.9%) vs. 2 (2.9%) and 6 (8.7%); *p* = 0.008, *p* = 0.195]. Similarly, the 3-year mortality rate was significantly higher in the upper SII tertile compared to the lower and middle tertiles [21 (30.4%) vs. 5 (7.1%) and 12 (17.4%); *p* < 0.001, *p* = 0.072]. The 5-year mortality rate was also significantly higher in the upper SII tertiles compared to the lower and middle tertiles [26 (37.7%) vs. 8 (11.4%) and 15 (21.7%); *p* < 0.001, *p* = 0.040]. Orhan *et al*. [37] consecutively enrolled 314 elderly patients with NSTEMI patients and divided them into three groups based on SII levels, designated as T1, T2, and T3. In-hospital and long-term mortality were defined as the primary outcomes. During the follow-up period, patients in the T3 group (indicating high SII level) had lower in-hospital and long-term survival rates compared to the T2 and T1 groups. A multivariate Cox regression model revealed that SII was independently associated with in-hospital mortality (hazard ratio [HR]: 1.001, 95% CI: 1.000–1.1003, *p* = 0.038) and long-term mortality (HR: 1.004, 95% CI: 1.002–1.006, *p* < 0.001).

Additionally, a study conducted by Karakayali *et al*. [38] included patients with ischemia with non-obstructive coronary arteries (INOCA). These patients presented with typical angina-like chest pain, had a normal resting 12-lead electrocardiogram, and showed positive results in exercise testing or myocardial perfusion imaging indicative of ischemia, despite having normal coronary angiography. The study found that a high SII level is independently associated with the presence of INOCA. SII could serve as a complementary indicator to traditional, expensive predictive methods for INOCA. The optimal cutoff value of SII for predicting INOCA was identified as 153.8, with a sensitivity of 44.8% and a specificity of 78.77% (area under curve: 0.651 [95% CI: 0.603–0.696, *p* = 0.0265]). It is now widely recognized that INOCA patients experience microvascular dysfunction, which is closely related to inflammation. Inflammation can contribute to the early onset of microvascular dysfunction in the initial stages of atherosclerotic lesions. While the study by Karakayali *et al*. [38] focused on INOCA patients, its objective was similar to ours—aiming to predict pathological changes before the onset of myocardial infarction and implement timely interventions to improve patient outcomes.

However, there are currently no studies focusing on ACS patients with UA. Our study included patients with concurrent DM, to further clarify the impact of inflammation levels on their prognosis. Ultimately, we also found that high inflammation levels are associated with poor prognosis in this patient group. After adjusting for traditional risk factors, we still found that higher levels of SII are related to long-term MACCEs events in UA patients with concurrent DM (*p* = 0.001, HR: 0.155, 95% CI: 0.063–0.382). This may suggest that we should enhance the differentiation of risk stratification for patients with different types of cardiovascular diseases. By implementing personalized treatment plans and strengthening the control of inflammation levels, we may potentially reduce the occurrence of complications.

Aside from the SII, many studies have identified various biomarkers that may be associated with poor prognoses, such as lipoprotein a (Lp a), hsCRP, interleukin (IL)-6, IL-1β, IL-1 receptor antagonist, and lipoprotein-associated phospholipase A2 [39]. For example, treatment with recombinant human IL-1 receptor antagonist anakinra has been linked to reduced mortality and heart failure risk in patients with STEMI, primarily by improving long-term outcomes through the inhibition of IL-1 activity [40]. Furthermore, elevated Lp a levels may contribute to the chronic inflammatory process [41]. Zhang *et al*. [42] also found that elevated Lp a (≥50 mg/dL) combined with elevated hsCRP (≥2 mg/L) was independently associated with a significantly increased risk of cardiovascular disease (HR: 1.62; 95% CI: 1.25–2.10) and all-cause mortality (HR: 1.39; 95% CI: 1.12–1.72). However, our study did not find an association between elevated he-CRP and adverse outcomes (HR: 1.057; 95% CI: 0.965–1.157; *p* = 0.234). This discrepancy may be due to the more restrictive inclusion criteria of our study population, particularly the inclusion of DM patients. In contrast, Zhang *et al*.’s study [42] included individuals from various ethnic backgrounds, all of whom were asymptomatic and free of clinical cardiovascular disease. Therefore, future large-scale randomized controlled trial (RCT) studies may further explore the predictive ability of factors such as Lp a in DM patients with coexisting UA.

## 5. Limitations

The primary limitation of this study was its single-center observational design. Despite using multivariable analysis, there may still be some unmeasured confounding factors that could affect the study results. Additionally, we calculated SII only once at admission and did not monitor changes in SII during the study period. Our retrospective approach also imposes limitations in terms of selection bias, information bias, and challenges in controlling confounding factors, which makes it difficult to infer clear causal inferences. Although our study initially excluded patients who were using antibiotics or had an active infection, we did track their medication during the follow-up period. Therefore, the potential impact of medication use on our results. Additionally, we included patients diagnosed with UA and diabetes at our center. The total number of cases is relatively small, and large-scale prospective studies are still needed to further clarify the prognosis.

## 6. Conclusion

For patients with UA combined with DM, elevated SII levels following PCI are significantly associated with adverse clinical outcomes, especially increased risks of MACCEs. This finding underscores the critical role of systemic inflammation in the progression of cardiovascular disease within this high-risk population. Regular monitoring of SII and other inflammatory biomarkers, such as hsCRP and IL-6, may provide valuable insights into the inflammatory status of these patients. Furthermore, implementing targeted strategies to control inflammation- such as optimizing glycemic control, utilizing anti-inflammatory medications, and promoting lifestyle modifications (e.g., weight management, smoking cessation, and regular physical activity), could potentially mitigate the inflammatory burden and improve long-term prognosis. Integrating these approaches into a comprehensive management plan may not only reduce the risk of recurrent cardiovascular events but also enhance overall patient outcomes. Further prospective studies are warranted to establish standardized protocols for inflammation monitoring and to evaluate the efficacy of anti-inflammatory therapies in this specific patient population.

## Availability of Data and Materials

Data can be made available upon reasonable request.
